# The loss of epigenetic information: not only consequences but a cause of mammalian aging

**DOI:** 10.1038/s41392-023-01412-9

**Published:** 2023-03-27

**Authors:** Chen Pan, Fangfang Zhou, Long Zhang

**Affiliations:** 1 International Biomed-X Research Center, Second Affiliated Hospital of Zhejiang University School of Medicine, Zhejiang University, Hangzhou, 310058 China; 2 Institutes of Biology and Medical Science, Soochow University, Suzhou, 215123 China; 3 Present Address: MOE Laboratory of Biosystems Homeostasis & Protection and Innovation Center for Cell Signaling Network, Life Sciences Institute, Zhejiang University, Hangzhou, 310058 China

**Keywords:** Ageing, Epigenetics

In a recent study published in *Cell*, Yang et al. revealed that changes in epigenetic landscapes caused by faithful DNA repair are key drivers accelerating aging of mammalian organs or tissues.^1^ Impressively, changes in H3K27ac landscape during aging process influence cell identity maintenance, and this aging process can be reversed by the inducible expression of pioneer transcription factors, Oct4, Sox2, and Klf4 (OSK) in the living mammals.

In mammals, global and local changes of DNA methylation occur in the genome during aging. Additionally, general loss of histones and global chromatin remodeling have been observed in all aging models, while in reverse reprograming of cell fate can lead to global changes in the epigenetic and rejuvenated epigenome, suggesting the potential of reprogramming for the reversal of aging.^2^ However, as no systematic studies revealed the characteristics of epigenomic changes during aging, it remains unclear whether the changes in epigenetic landscape are the consequences (marks) or direct cause of aging. The authors previously found that the loss of epigenetic landscapes during aging is driven by responses to cellular damage, such as DNA double strand breaks (DSBs), and this is a conserved mechanism in eukaryotes from yeast to mammals, which provided a model to test the relationship between epigenomic changes and mammalian aging.

**Fig. 1:**
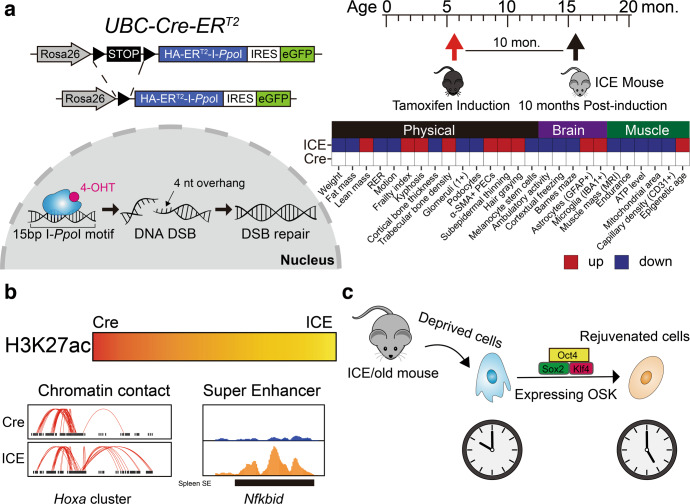
Diagram and molecular mechanisms underlying the ICE mouse models. **a** left, the Inducible Change to the Epigenome (ICE) system. In this gene-engineering mouse model, recombined protein of endonuclease I-*Ppo*I and Cre-ER^T2^ is under control of tamoxifen. If tamoxifen is added, I-*Ppo*I will enter nucleus to create DNA double strand break which then will be repaired through faithful DNA repair pathway. right, aging phenotypes of ICE models. **b** Epigenetic landscapes changes in ICE model, including the erosion of H3K27ac, remodeling chromatin contact, and aberrant super enhancers. **c** The diagram of cell rejuvenation. Briefly, overexpression Oct4/Sox2/Klf4 in ICE/old mouse cells can recover youthful cell transcriptome

The authors engaged a transgene mouse model (termed Inducible Changes to the Epigenome (ICE) system or ICE mice), in which the I-PpoI restriction enzyme targets 20 canonical sites (19 are at non-coding regions) in the mouse genome and creates DSBs with a low mutation rate under the control of tamoxifen (Fig. 1a). At 10 months post-induction, they observed that ICE mice exhibited physical aging (Fig. 1a). Moreover, post-induction ICE mice presented brain and muscle aging as indicated by multiple analyses showed in Fig. 1a. As the level of CpG site methylation is a reliable biomarker for predicting age, the authors established two epigenetic clocks, adapting the Elastic Net regression model to calculate the epigenetic age of post-induction ICE mice based on age-associated CpGs for blood (743) and muscle (2048). According to these two clocks, ICE mice exhibited a 50% faster rate of aging. In conclusion, this study provided a new accelerated aging mouse model, which physically and epigenetically phenocopied the normal mouse aging process.

To mechanistically address the reasons for the faster aging of ICE mice, the authors carried out mass spectrometric quantification and ChIP-seq experiments of multiple histone modifications, and post-treated ICE cells showed lower levels of H3K27ac signals, corresponding to a genome-wide erosion of H3K27ac landscape (Fig. 1b). Interestingly, the homeobox (*Hox*) cluster, a developmental transcription factor family, was characterized by altered H3K27ac, H3K56ac, and H3K27me3 signals. In addition, the spatial organization of chromatin in post-induction ICE cells was also altered at the *Hox* site, firstly providing evidences that multiple regulatory mechanisms existed in cell aging, such chromatin modifications and chromatin contacts, are driven by faithful DNA repair (Fig. 1b). In mammals, H3K27ac signals-marked enhancers or super enhancers (SEs) are linked to cell identity.^3^ In post-induction ICE mice, organ-specific SEs were dysregulated, for example, the H3K27ac signals at spleen-specific SE of *Nfkbid* were abnormally enhanced in muscle, indicating that the muscle of post-treated ICE mice lost their original identity and ex-differentiated toward an immune signature (Fig. 1b). Intriguingly, the forced expression of Oct4, Sox2, and Klf4, which are pioneer transcription factors of mouse embryonic stem cells, rejuvenated the mRNA transcriptome of cells from posted-induction ICE mice or older mice (Fig. 1c), corroborating that epigenomic changes constitute one of the causes of the aging process.

Many factors affect the aging process and longevity, including telomere shortening, mitochondrial dysfunction, and over-intake of nutrients, based on which aging accelerated mouse models have been constructed in the past years.^4^ Compared with the D-galactose-induced senescence model or Total body irradiation (TBI) model, the ICE model has several advantages. First, mammalian cells suffer from DNA repair stress during cell proliferation and differentiation. Instead, excessive intake of nutrients or irradiation are not stresses faced by major mammalian populations. Second, the ICE model is more directional and robust as irradiation results in side effects, such as leukemia or solid tumors. While irradiation directly leads to DNA DSBs, it is associated with higher rates of error-prone repair and mutations; instead, the ICE model prefers the faithful DNA repair. Third, the ICE model is an inducible system that can be modified to function in tissues or organs of interest, thus enabling the exploration of the connections between different tissues or organs during the aging process. The limitations of this study were that no deep analysis was performed, such as building single-cell transcriptome atlas or epigenome profiling.

As a result of post-induction in the ICE model, huge changes occurred in the epigenome, especially global erosion of one histone modification-H3K27ac that helps verify the enhancers in each cell lines. As super enhancers are considered regulators of cell identity or fate in epigenome, the findings of this study were in line with the concept that super enhancers control cell identity during the aging process. Accordingly, epigenomic changes during the aging process cause loss of cell identity, leading to the defects in some tissues or organs. Additionally, the conclusion also helps explain why cell reprogramming via induced expression of OSK can rejuvenate aging cells. Apart from DNA demethylation, H3K27ac reprogramming dominated by OSK is a key step to rejuvenate aged cells.^5^ Hence, this study implied small molecular or compounds inducing cell reprogramming can be further tested for rejuvenating aging cells in future studies.

Overall, Yang et al. provided a fundamental basis for studying mammalian aging process, and even rejuvenate living organisms in the foreseeable future.
